# Improvement of Disease Prediction and Modeling through the Use of Meteorological Ensembles: Human Plague in Uganda

**DOI:** 10.1371/journal.pone.0044431

**Published:** 2012-09-14

**Authors:** Sean M. Moore, Andrew Monaghan, Kevin S. Griffith, Titus Apangu, Paul S. Mead, Rebecca J. Eisen

**Affiliations:** 1 National Center for Atmospheric Research, Boulder, Colorado, United States of America; 2 Centers for Disease Control and Prevention, Division of Vector-Borne Diseases, Fort Collins, Colorado, United States of America; 3 Uganda Virus Research Institute, Arua, Uganda; Public Health Ontario, Canada

## Abstract

Climate and weather influence the occurrence, distribution, and incidence of infectious diseases, particularly those caused by vector-borne or zoonotic pathogens. Thus, models based on meteorological data have helped predict when and where human cases are most likely to occur. Such knowledge aids in targeting limited prevention and control resources and may ultimately reduce the burden of diseases. Paradoxically, localities where such models could yield the greatest benefits, such as tropical regions where morbidity and mortality caused by vector-borne diseases is greatest, often lack high-quality *in situ* local meteorological data. Satellite- and model-based gridded climate datasets can be used to approximate local meteorological conditions in data-sparse regions, however their accuracy varies. Here we investigate how the selection of a particular dataset can influence the outcomes of disease forecasting models. Our model system focuses on plague (*Yersinia pestis* infection) in the West Nile region of Uganda. The majority of recent human cases have been reported from East Africa and Madagascar, where meteorological observations are sparse and topography yields complex weather patterns. Using an ensemble of meteorological datasets and model-averaging techniques we find that the number of suspected cases in the West Nile region was negatively associated with dry season rainfall (December-February) and positively with rainfall prior to the plague season. We demonstrate that ensembles of available meteorological datasets can be used to quantify climatic uncertainty and minimize its impacts on infectious disease models. These methods are particularly valuable in regions with sparse observational networks and high morbidity and mortality from vector-borne diseases.

## Introduction

Climate variation and weather patterns have been linked to the occurrence of a number of infectious diseases [1]. Pathogens causing zoonotic (e.g. hantaviruses or plague), and vector-borne diseases (e.g. malaria, dengue, tick-borne encephalitis, Lyme disease), are particularly sensitive to meteorological variables such as temperature and precipitation because these variables affect vector and host population dynamics in addition to pathogen transmission [1], [2], [3]. Capitalizing on these relationships, weather and climate variables have been used successfully to model the spatial and temporal distributions of several vector-borne and zoonotic diseases [4], [5], [6], [7]. Understanding how climate and weather influence disease occurrence in a particular geographic region is an essential part of disease forecasting, and can help promote the timely implementation of disease control and prevention efforts [8]. A better understanding of the association between weather patterns and disease occurrence is also a necessary step in determining how climate change may affect the distribution and incidence of different infectious diseases [1], [2].

The per-capita mortality, morbidity, and economic burden associated with vector-borne diseases are highest in tropical and sub-tropical regions including sub-Saharan Africa and Southeast Asia [9], [10], [11]. Furthermore, the emergence of new vector-borne and zoonotic infectious diseases is also most likely to occur in the tropical regions of Africa and southern Asia [12]. However, because a large proportion of tropical countries are underdeveloped [13], the tropics also have the sparsest coverage of quality-controlled, ground-based meteorological data [14]. In areas where meteorological stations are absent, it is necessary to use satellite- or model-based gridded regional or global climatologies to explore the association between climate or weather and the occurrence of infectious diseases. Several different datasets are available for Africa, however they vary in their spatial resolution as well as their ability to accurately resolve weather patterns, particularly in areas with complex topography [15], [16], [17]. The problem of having sparse or incomplete meteorological data for examining associations between disease and climate is not new, but has been overcome in previous studies of historical disease outbreaks in innovative ways. For example, in lieu of traditional climate records, the paleoclimatic forcing of plague in Central Asia over the past millennium has been demonstrated using several climate proxy data sources (glacial ice cores, tree rings, and stalagamite isotope data) that associate major human plague outbreaks with periods favorable for epizootics in the wild rodent hosts of the bacteria [18], [19].

The selection of datasets to be used as independent variables for disease forecasting models influences the ability of the models to detect an association between weather and disease occurrence, as well as the functional relationship between the meteorological variables and the disease outcome. Due to uncertainty in individual gridded meteorological datasets that arises for various reasons (e.g., poor constraint from in situ observations, coarse spatial and temporal scales, algorithm assumptions, etc.), techniques that employ only one realization of a meteorological variable (i.e., one dataset) to develop and implement a disease model may not adequately capture the range of possible weather (and therefore disease) outcomes, especially in data-sparse regions like the tropics [20], [21], [22]. In such cases, employing ensembles of gridded meteorological datasets to develop and implement disease models may provide more robust results for weather-sensitive disease outcomes, as well as measures of uncertainty. We use plague in the West Nile region of Uganda to evaluate how selection of meteorological variables and use of ensemble and model averaging techniques influences plague model outcomes, specifically the inter-annual variation in case counts.

Plague is a rodent-associated flea-borne disease caused by the bacterium *Yersinia pestis*. Plague has caused three major human pandemics that killed millions. Although improved sanitation and access to antibiotics has reduced disease incidence and case fatality rates, sporadic cases and focal outbreaks still occur regularly. The majority of human cases in the last few decades have occurred in eastern and southern Africa [23], [24], [25], where case fatality rates may be as high as 40% [26]. Outcome of infection can be improved by early diagnosis and treatment with antibiotics [27], [28]. A system forecasting the expected incidence of plague in a particular region during the next year would help inform local public health officials, health care providers, and the general public, and better utilize resources for prevention and control efforts.

Previous models have identified strong associations between temperature and precipitation and plague occurrence [4], [19], [29], [30], [31], [32], [33], [34]. However, the majority of these studies were focused in temperate regions that show a clear seasonality and where human cases are rare. By contrast, similar models for tropical areas, such as plague foci in Africa, are uncommon. Here, we examine the relationship between regional temperature and rainfall and the inter-annual occurrence of human plague in the West Nile region of northwestern Uganda from January, 1999 to July, 2011. Because ground-based meteorological data in this region is very limited, we used several different publicly available gauge-, model- and satellite-derived gridded climate and meteorological datasets from U.S. and European data archives. First, we used the collection of meteorological datasets to create an ensemble dataset based on the estimated accuracy of the different datasets within our study region. Next, we used the meteorological ensemble dataset to evaluate how well meteorological data described variation in historical inter-annual plague occurrence. Finally, we assessed the sensitivity of model results to the selection of different datasets using model averaging techniques. We show that by incorporating both within-dataset and between-dataset variation and uncertainty, the modeled association between climate and plague occurrence is less sensitive to biases and errors associated with an individual data source. Model selection identified several rainfall variables that are strongly associated with the annual number of human plague cases.

### Study area background

**Figure 1:**
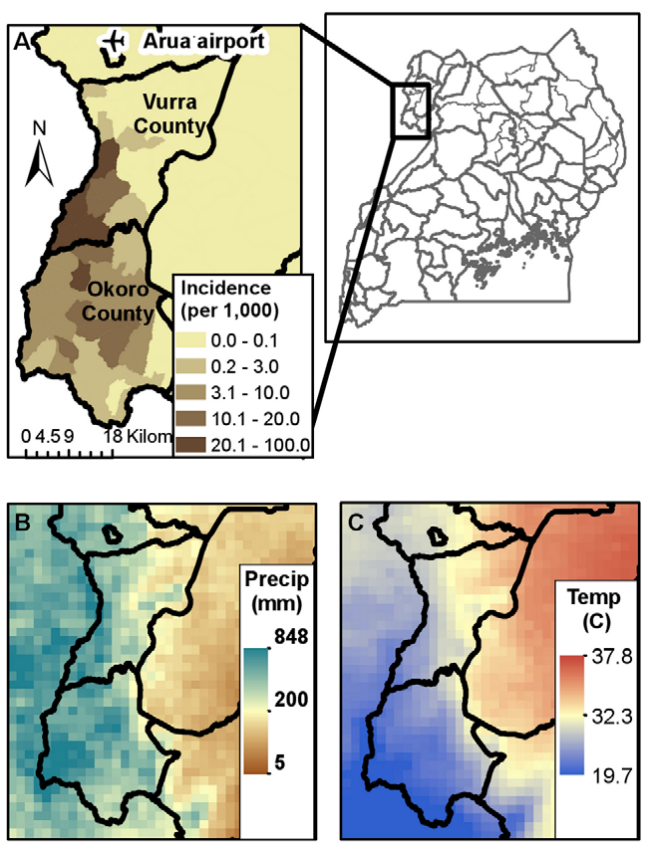
Spatial distributions of plague, temperature, and rainfall in West Nile region of Uganda. (A) Reported cumulative plague incidence per 1,000 population from 1999–2007 in Vurra and Okoro counties of Uganda. (B) Average August rainfall (mm) and (C) average February maximum temperatures (°C) in northwestern Uganda. Temperature and rainfall averages were based on data from 1999–2009 generated using a 2 km Weather Research Forecasting (WRF) model [68].

**Figure 2:**
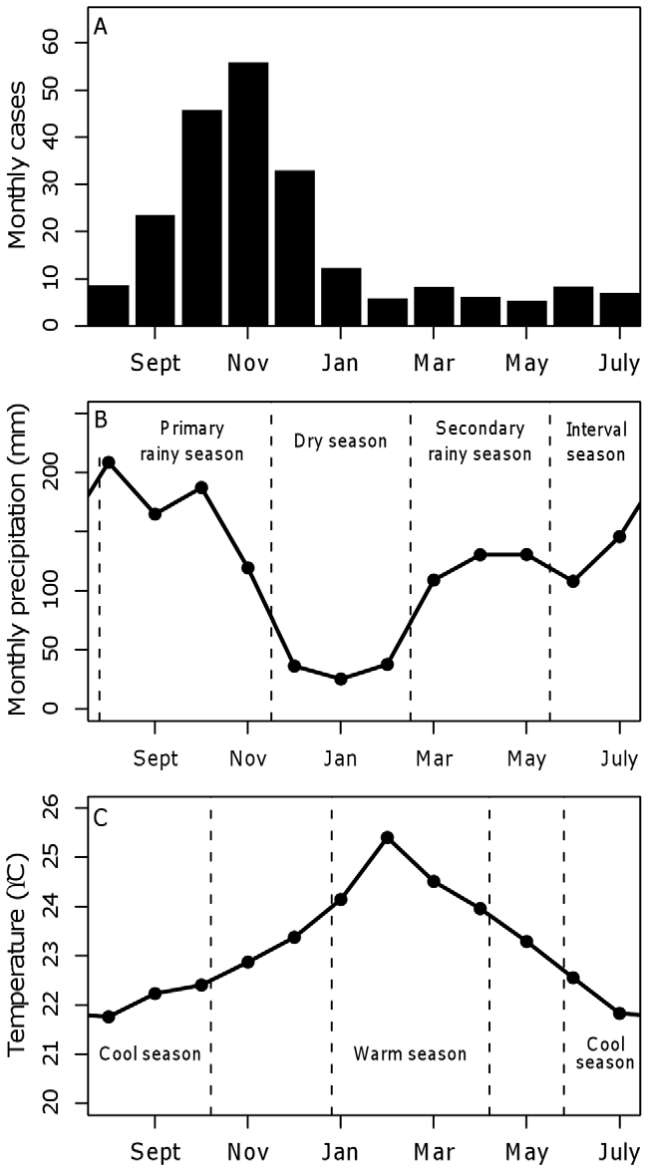
Seasonal patterns of suspect plague cases, temperature, and rainfall. Average monthly (A) number of suspect plague cases, (B) rainfall, and (C) mean temperature in Vurra and Zombo counties, Uganda for 1999–2010. Data are organized by plague year which runs from August to July. Temperature data are averages of monthly means from the Arua airport observational dataset and the ERA-Interim dataset. The warm season is from January to April and the cool season is from June to October. Rainfall data are average monthly rainfall totals from the CMORPH, TRMM, FEWS-Net, and the Arua airport observational datasets. (See text for further details regarding the meteorological datasets and seasonal descriptions).

**Figure 3:**
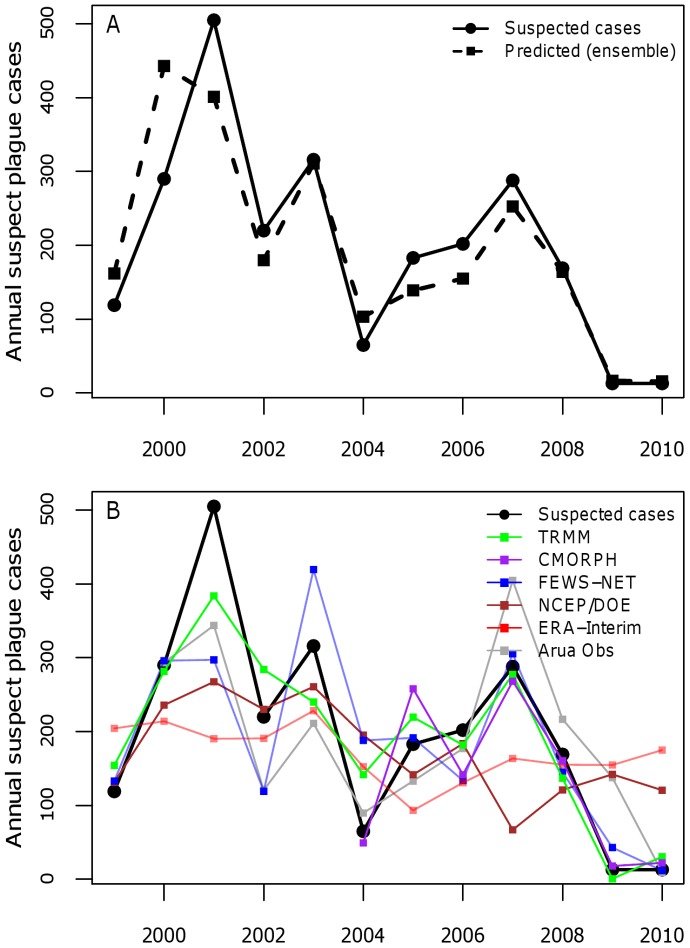
Annual number of observed versus predicted suspect plague cases. (A) Plot of annual number of observed vs. predicted number of suspect human plague cases from the best-fit regression model using the meteorological ensemble dataset that uses a weighted average of all of the rainfall and temperature datasets included in this study (see Table 2 for model details). (B) Predicted number of suspect human plague cases from the best-fit regression model using each of the individual rainfall datasets.

Plague cases in Uganda are concentrated within Okoro and Vurra counties in the West Nile region (Figure 1a). These two counties are located on the Rift Valley escarpment in the districts of Arua (Vurra) and Zombo (formerly Nebbi) and are bordered on the west by the Democratic Republic of Congo (DRC). Plague is highly seasonal in Uganda, with the majority of cases typically arising between September and December each year (Figure 2a). For the purposes of our analysis, we defined a plague year as August of one year to July of the following calendar year. A total of 2,409 suspect plague cases were reported in Okoro and Vurra counties from August, 1999 to July, 2011, a mean of 201 cases per year for both counties combined. The number of annual suspect cases ranged from a high of 505 during the 2001–2002 plague season to a low of 13 during the 2009–2010 and 2010–2011 plague seasons (Figure 3a). There was a non-significant negative linear trend in the number of annual plague cases over the time period of this study (F_1,10_ = 3.847; p = 0.08).

The higher elevations in Okoro and Vurra counties experience lower temperatures and more annual rainfall than in the Nile Valley east of the escarpment (Figure 1b,c). Rainfall in the region falls mainly during a secondary rainy season from March to May, followed by a primary rainy season from July/August through November (Figure 2b). Monthly variations in temperature are relatively minor, with mean temperatures ranging from 21.8°C in August to 25.4°C in February (Figure 2c).

## Results

### Development of meteorological ensembles

**Figure 4:**
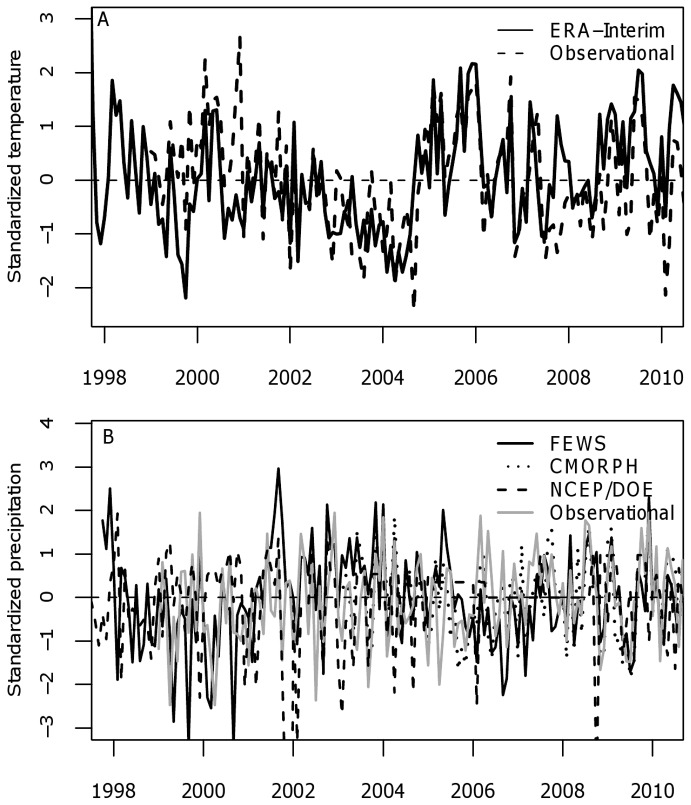
Variability among the different temperature and rainfall data sources. (A) Standardized monthly mean temperatures (1998- present) from the Arua airport (Observational) and ERA-Interim datasets. (B) Standardized monthly days of rainfall >0.2 mm (1998-present) from Arua airport (Observational), National Centers for Environmental Prediction re-analysis II project (NCEP/DOE), USAID Famine Early Warning System Network (FEWS-Net), and Climate Prediction Center morphing technique (CMORPH) rainfall datasets. The other rainfall datasets were not included in the figure to maintain clarity.

Good quality ground-based meteorological data for the northwestern highlands region of Uganda is largely non-existent. There are no publicly available meteorological datasets from Vurra or Okoro counties. We employed meteorological data from the closest station with daily temperature and rainfall observations for the time period of this study, the airport in Arua, Uganda, which is located several kilometers north of Vurra County (see Figure 1a) at an elevation of 1204 m, below the 1300 m threshold for high plague risk [35], [36]. We also selected one additional temperature dataset and six additional gauge- and satellite-estimated or re-analysis rainfall datasets based on their estimated accuracy in sub-Saharan Africa (Table 1; SI Materials and Methods; Figure 4).

**Table 1:** Meteorological datasets used in analysis of the interannual variation of suspect plague cases.

Dataset	Source	Time span	Spatial resolution	Ensemble weight	Reference
*Temperature*					
Arua airport	Local meteorological station	1/1999–12/2010	N/A		-
ERA-Interim	European Centre for Medium-Range Weather Forecasts - Interim reanalysis project	1/1998–12/2010	1.5° lat./long.		[70]
*Rainfall*					
Arua airport	Local meteorological station	1/1999–12/2010	N/A	0.143^1^	-
ERA-Interim	European Centre for Medium-Range Weather Forecasts - Interim reanalysis project	1/1998–12/2010	1.5° lat./long.	0.065	
NCEP/DOE	National Centers for Environmental Prediction Reanalysis II project	1/1998–12/2010	1.875° lat./long.	0.066	[71]
GPCP	Global Precipitation Climatology Project	1/1998–4/2008	1° lat./long.	0.133	[72]
TRMM	Tropical Rainfall Measurement Mission -Multisatellite Precipitation Analysis project, product 3B42	1/1998–12/2010	0.25° lat./long.	0.179	[73]
CMORPH	National Oceanic and Atmospheric Administration Climate Prediction Center - Morphing Technique project	1/2003–12/2010	0.07° lat./long.	0.123	[74]
FEWS-NET	United States Agency for Development - Famine Early Warning System Network	1/1998–12/2010	0.1° lat./long.	0.265	[75]

Time span is the period of the analysis for which each dataset is available. Spatial resolution is represented by decimal degrees of latitude and longitude. Ensemble weight is the weight each individual dataset was given in the ensemble rainfall dataset based on ground-truthing. The last column provides a reference for additional information on each dataset.

1 Initial ensemble weight of 0.143 for Arua airport rainfall dataset was set at 0.143 (1/7) because dataset could not be ground-truthed.

To develop a meteorological ensemble, we first evaluated the ability of the six gauge- and satellite-estimated datasets to reproduce observed seasonal rainfall variability measured at all rain gauges within 500 km of Okoro and Vurra counties. The FEWS-Net rainfall dataset had the highest mean correlation (r = 0.34) when compared on a site-by-site basis with the normalized seasonal rainfall frequencies from the 11 meteorological stations within the 500 km radius. The TRMM (r = 0.23), GPCP (r = 0.17), and CMORPH (r = 0.16) rainfall datasets had intermediate correlation values, while the two re-analysis datasets, ERA-Interim and NCEP/DOE—which have comparatively coarse spatial resolutions–were poorly correlated (r = 0.08) (Table S2). Based on these correlations FEWS-Net was given the highest weighting in the rainfall ensemble dataset (see Table 1 for weights). The Arua airport rainfall observations – which have interannual variations that are representative of the broader regional variability over Vurra and Okoro counties – were given a ‘neutral’ weight of 0.143 (1/7). The airport data, despite being the only ‘true’ *in situ* observational data among the 7 rainfall datasets, were not given greater weighting because of uncertainty arising from collection techniques, transcription errors, and missing data. The ERA-Interim and NCEP/DOE re-analysis rainfall datasets were included in the weighted ensemble, but excluded from the model averaging of individual dataset results (see description of technique below) due to their poor correlations with surrounding meteorological stations. The temperature ensemble dataset was created by averaging the ERA-Interim and Arua airport datasets with equal weights. Modeled and measured temperature datasets are generally of much higher quality than rainfall datasets, and therefore uncertainty among datasets is not as big of a concern for temperature.

### Association between weather and plague occurrence

**Figure 5:**
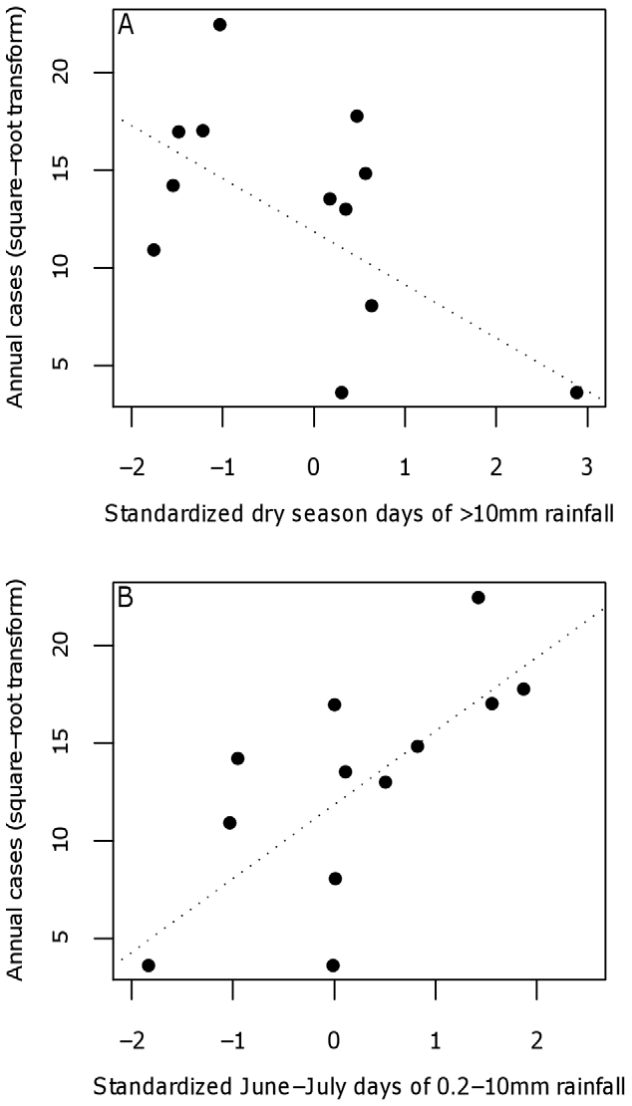
Association between rainfall and plague occurrence. Relationship between square-root transformed number of annual suspect plague cases and (A) the standardized number of days of >10 mm in the dry season (December-February) prior to the start of the plague year in August (zero-year lag) and (B) the standardized number of days of 0.2–10 mm rainfall in June and July prior to the start of the plague year (zero-year lag). The rainfall data is the weighted ensemble of all seven rainfall datasets included in our analyses (TRMM, CMORPH, FEWS-NET, NCEP/DOE, ERA-Interim, GPCP, and Observational). Dotted lines are the regression coefficients estimates from the best two-variable model using the ensemble rainfall dataset.

The best fit model to the inter-annual case data with the weighted meteorological ensemble dataset (AICc = 19.6, adjusted r^2^ = 0.864; Figure 3a) contained a negative association with the number of days of >10 mm of rainfall in the preceding dry season (December-February) (Figure 5a) and a positive association with the number of days with between 0.2–10 mm of rainfall during the interval between rainy seasons from June to July prior to the start of the plague season (Figure 5b). Including interactions or quadratic terms of the significant predictors did not improve model fit, and the number of suspect plague cases in the previous year was not a significant predictor when included in the model (p>0.20). No other model had a ΔAICc<2 (Table 2). The model selected with the weighted meteorological ensemble dataset performed better than a model with the same variables using an unweighted ensemble dataset (adjusted r^2^ = 0.864 vs. 0.719). The leave-one-out method indicated the best fit model was not overly sensitive to input from any one year (mean R^2^ = 0.861, 95% CI: 0.796–0.926). In addition, the weighting of the observational rainfall dataset from Arua in the rainfall ensemble dataset did not influence results; the best fit model was identical when the weighting was varied from 0% to 50%.

**Table 2:** Model AICc, ΔAICc, and adjusted r^2^ values for the best fit models (all models with ΔAICc<10) using the weighted meteorological dataset ensemble with square-root transformed number of suspect plague cases as the response variable.

Coefficient #1	Coefficient #2	AICc	ΔAICc	Adj. R^2^
>10 mm Dry season rainfall (−)	0.2–10 mm June/July rainfall (+)	19.6	0	0.864
>2 mm Dry season rainfall (−)	0.2–10 mm June/July rainfall (+)	23.1	3.4	0.819
>2 mm Dry season rainfall (−)	>2 mm June/July rainfall (+)	25.3	5.7	0.781
>10 mm Dry season rainfall (−)	>2 mm June/July rainfall (+)	29.3	9.6	0.696

ΔAICc represents the difference between a model's mean AICc value and the mean AICc value of the best fit overall model. +/− symbol after the coefficient names indicates whether the coefficient value is positive or negative.

To test the robustness of the best-fit regression model generated using the weighted meteorological ensemble dataset to variability between individual meteorological datasets, we determined the best-fit model using each of the 18 different temperature and rainfall dataset combinations, and then averaged model statistics and parameter values across all 18 combinations. Among the potential models predicting the square-root transformed number of annual suspect plague cases for each of the 18 dataset combinations, 63 two-variable models, but no single variable models, had ΔAICc<2. The mean adjusted r^2^ across all 18 potential meteorological datasets was highest for the models with the number of days of between 0.2 and 10 mm of rainfall during June-July and either the days of >2 mm (Model #1) or >10 mm (Model #2) of rainfall during the preceding dry season as the explanatory variables (mean r^2^ = 0.65 and 0.62; Table S3). The difference between the mean adjusted r^2^ values for these two models was not statistically significant (one-sided, paired Student's t-test; t = 1.222, p = 0.12) and the mean ΔAICc between the two models was only 1.8, indicating significant support for either consensus model. No other models had a mean ΔAICc<2 when model results were averaged across all potential meteorological datasets. Both explanatory variables were statistically significant in each of the two consensus best two-variable models with a ΔAICc<2 (Table S3). In each model, the number of days with between 0.2 and 10 mm of rainfall in June and July was positively associated with the annual number of the suspect plague cases at a zero-year lag (i.e., wetter-than-normal conditions in the interval rainy season immediately prior to the plague season). In addition, the number of suspect plague cases was negatively associated with the number of days in the dry season with either >2 mm or >10 mm of rainfall at a zero-year lag (i.e., more cases following drier-than-normal conditions during the preceding December-February dry season).

There was large variability in model fit when the consensus best two-variable models were run using the individual rainfall datasets (Figure 3b). Adjusted r^2^ values ranged from a high of 0.87 with the CMORPH data (which only covers 2003–2010) to a low of <0 (un-adjusted r^2^<0.15) using the coarse-resolution ERA-Interim or NCEP/DOE data for the model with >2 mm of dry season rainfall and between 0.2–10 mm of June-July rainfall as parameters (Table 3). Both variables from the consensus best two-variable model were statistically significant for only two of the seven rainfall datasets (TRMM and FEWS-NET), and neither were significant for three of the seven datasets, which happen to have the coarsest spatial resolution (GPCP, ERA-Interim, and NCEP/DOE). The large range of results shown in Table 3 and Figure 3b underscores the importance of performing quality control on meteorological datasets prior to using them, and of employing ensemble approaches likes the one used here for disease model development and implementation, to increase the likelihood of robust results and to understand model uncertainty.

**Table 3:** Model adjusted r^2^ values and coefficient estimates for the two consensus best-fit models when run using the meteorological ensemble dataset and each of the individual precipitation datasets.

	Model #1			Model #2		
Precipitation Data set	Adj. R^2^	Dry season >2 mm rain	0.2–10 mm June–July rain	Adj. R^2^	Dry season >10 mm rain	0.2–10 mm June–July rain
Arua Obs.	0.48	−2.72	NS	0.55	−2.73	NS
CMORPH	0.87	−2.36	NS	0.70	−2.62	NS
TRMM	0.77	−1.87	1.99	0.83	−2.34	2.05
FEWS-NET	0.84	−2.26	1.76	0.66	−2.09	2.94
GPCP	0.13	NS	NS	0.12	NS	NS
*ERA-Interim*	−0.14	NS	NS	−0.12	NS	NS
*NCEP/DOE*	−0.09	NS	NS	0.01	NS	NS
Ensemble	0.82	−2.31	2.07	0.86	−2.72	3.78
**Model means**	0.65	−1.95	1.76	0.62	−2.05	2.19

Coefficients estimates not significant at the α = 0.05 level are listed as NS. Model mean values represent the mean adjusted r^2^ and coefficient estimates averaged across model results from the individual and weighted ensemble meteorological datasets (results from the ERA-Interim and NCEP/DOE datasets were not included in the model means due to their low accuracy in the study region). All model mean coefficients were significant at the α = 0.05 level.

## Discussion

In areas where meteorological stations are absent, satellite-derived and global climate-reanalysis datasets can be used to address the link between weather and disease occurrence for explanatory or predictive purposes. However, as we have shown here, the correlation between different datasets is not always strong, and for many regions it is difficult to assess which datasets are most accurate. To address this issue we generated a suite of descriptive models using several different temperature and rainfall datasets and tested these models with ensemble datasets. Model fit varied among datasets, but the two key rainfall variables, 0.2–10 mm June-July rainfall and >10 mm (or >2 mm) dry season rainfall, remained significant even when their effects were averaged across all datasets. Using multiple meteorological or climatological datasets and model-averaging techniques provides a conservative approach that reduces the possibility of detecting a spurious correlation, or failing to detect an actual correlation, between disease occurrence or incidence and variables from a single dataset that may not be highly accurate in a particular study area.

Ensembles of meteorological or climatological models are frequently used to reduce the uncertainty inherent in probabilistic weather and climate forecasting because regional and global climate models vary considerably in their ability to accurately represent different aspects of weather or climate dynamics [20], [21], [22], [37], [38]. Multi-model ensemble averages are better at representing current climate conditions and past changes than any single global circulation model for a range of climate variables [20], [37]. The high level of variability between rainfall frequencies in the West Nile region of Uganda for the different gauge- and satellite-derived meteorological datasets included in this study indicates that it is also important to address the uncertainty in gridded meteorological data when attempting to determine the response of ecological processes, such as infectious disease occurrence, to weather patterns. Weighting the meteorological ensemble based on the degree of agreement with ground-based observational rainfall frequencies from local meteorological stations resulted in a strong model fit. However, even an unweighted ensemble of all seven rainfall datasets still produced a moderately strong model fit and both rainfall variables were statistically significant. This relatively good fit was partly because the unweighted rainfall ensemble dampened the effects of spurious rainfall values from any one member. Therefore, in situations where ground-truthing would be difficult or when rapid prediction is desired, using an unweighted multi-dataset ensemble would likely represent an improvement over the selection of a single meteorological dataset.

For three of the seven individual rainfall datasets considered in this study, neither of the rainfall variables from the consensus best two-variable model was statistically significant. These three rainfall datasets (GPCP, ERA-Interim, and NCEP/DOE) had the coarsest spatial resolution of the datasets considered in this study, suggesting that the use of these datasets may not be appropriate in topographically complex regions such as along the Rift valley in East Africa. It is therefore important to validate global or regional meteorological datasets using other data sources, such as regional meteorological stations as we have done here. The local rainfall data from the *in situ* Arua airport record only produced a moderate model fit (adjusted r^2^ = 0.48) and the standardized frequency of 0.2–10 mm June-July rainfall was not statistically significant for this dataset, despite consistently being the strongest predictor among other datasets. This suggests that caution be exercised when attempting to extrapolate regional meteorological conditions from a single data source, particularly if the quality of the data is uncertain and/or its location may not be representative of the entire study region. In this case, we believe the Arua airport record is reasonably representative of the broader regional rainfall and temperature variability (as informed by comparison of grid points in the gridded datasets), but that its quality (especially for the rainfall data) is questionable due to measurement uncertainty, transcription errors, and data outages. Although model fit with one or more of the individual rainfall datasets was comparable to model fit with the ensemble dataset for either of the consensus best two-variable models, no individual rainfall dataset performed as well as the ensemble dataset for both consensus models. The inability of any individual dataset to perform well with both models is likely due to differences between how strongly the frequencies of >2 mm and >10 mm rainfall are correlated for a given dataset based on a bias towards or against capturing heavier rainfall events. Because the individual rainfall datasets were compared with surrounding ground stations using the number of days with>0.2 mm rainfall, the dataset(s) that were identified as having the highest correlation with measured rainfall amounts might not be the best at representing the frequency of heavier rainfall events. Therefore caution should be exercised before selecting a single data source based solely on its correspondence with overall rainfall frequencies or amounts, without also considering its accuracy at representing the frequency of heavier rainfall events that might influence disease occurrence.

We found that the annual number of suspect plague cases in the West Nile region from 1999–2010 was negatively linked to the amount of rainfall during the December-February dry season and positively associated with rainfall in the June-July interval season immediately prior to the plague transmission season in August. Rainfall is an important determinant of the geographic distribution and timing of plague outbreaks in wildlife and humans [4], [19], [29], [30], [31], [32], [33], [39], [40]. The occurrence of bubonic plague in arid or semi-arid temperate regions is positively associated with the amount of annual or seasonal rainfall one or two years prior [4], [32], [34], [39]. In tropical regions the association with rainfall is mixed; plague intensity in southern China is positively associated with drought conditions during the preceding year and extremely wet or extremely dry conditions in the current year [34] and most human cases occur during the dry season in Vietnam [41], [42], [43]. However, in other tropical and sub-tropical regions plague generally occurs during the rainy season or shows no clear seasonal pattern [44], [45]. In addition, previous research in the West Nile region of Uganda established a positive link between rainfall in February, October, and November (but a negative association with June rainfall) and the spatial risk of plague [40].

The positive association between plague and lagged rainfall in arid and semi-arid regions has been explained by the trophic cascade hypothesis [4], [32]. Increased rainfall increases plant primary productivity, which leads to increases in rodent abundance. In several systems, plague epizootics in rodent populations, or an increased risk of spillover to humans, are associated with rodent populations surpassing a threshold density [46], [47], [48], [49], [50]. In the American Southwest the positive association between rainfall and plague occurs at a lag of one to two years, likely because there is a delay in the response of rodent abundance to increased primary productivity [4]. In our study, we found a positive association between rainfall in June and July and the number of cases in the immediately following August-July plague year. However we found no evidence of a positive association between plague occurrence and rainfall in the preceding years. The rodent found most often in and around human dwellings in the West Nile region of Uganda is the roof rat, *Rattus rattus*, and the most abundant sylvatic rodent is the Nile rat, *Arvicanthis niloticus* [51], [52]. Unlike the ground squirrels (*Spermophilus* and *Amerospermophilus* spp.) and prairie dogs (*Cynomys* spp.) that are believed to be the primary epizootic hosts in the American Southwest and have relatively low reproductive rates [4], *R*. *rattus* can produce up to five litters of 6–12 young per year and *A. niloticus* is capable of giving birth to litters of 4–12 young every 23 days during the breeding season [53]. In addition, both species can breed year-round under highly favorable conditions. Due to the high reproductive rates of these species, we might therefore expect a much more rapid response to changes in rainfall and other environmental conditions.

The association between rainfall and plague occurrence could also arise due to the influence of rainfall on vector and rodent ecology in a more complex manner than a direct trophic cascade. Higher rainfall may increase flea survival [32], and plague occurrence is positively associated with rodent flea burdens [43], [54], [55]. Therefore, higher rainfall in June and July could increase flea abundance, increasing the likelihood of plague transmission in subsequent months. In the West Nile region of Uganda, an elevated risk of plague occurrence at the individual hut level has been associated with proximity to annual crops including corn [56]. Drier dry seasons and wetter months of June and July (between the two rainy seasons) are likely to influence both crop productivity and the timing of planting and harvesting of these crops. Plague risk in this region has also been associated with the presence of bare ground in January when annual crop fields are typically fallow [35]. Less rainfall during the dry season from December through February may alter the timing of crop planting or it may influence rodent abundance and behavior. Human plague cases occur following epizootics and subsequent die-offs in the domestic-dwelling *R. rattus* population [52], but it is believed that *Y. pestis* is maintained long term in the sylvatic and peridomestic rodent populations (particularly *A. niloticus*) [44], [52], [57], [58], [59], [60], [61], [62]. Therefore an increase in human plague cases may result from conditions that promote epizootics in *R. rattus*, increase the prevalence of *Y. pestis* in sylvatic rodent populations, or increase the spillover rate from sylvatic rodent populations to *R. rattus*. The primary route of transmission between the sylvatic, peridomestic, and domestic settings in this region appears to be via contact between *R. rattus* and *A. niloticus* (Amatre et al. 2009). Further research is needed to determine how the contact rates between these rodent species varies seasonally and from year to year, and whether these changes are associated with variations in rainfall and associated factors such as the timing of annual crop harvests.

The model results presented here are descriptive only; we could not explicitly evaluate the causal relationship between rainfall and the annual variation in plague occurrence in the West Nile region of Uganda. Field studies are needed to examine how flea abundance, rodent abundance, and rodent contact rates are influenced by rainfall changes in the two time periods identified in this study. Research should also be conducted to determine how rainfall during these time periods influences primary productivity in the sylvatic environment and agricultural variables such as crop yield and the timing of planting, harvesting, and drying of annual crops such as corn. The predictive ability of the model is limited by the fact that we used human plague cases as a response variable. Without knowledge of rodent population dynamics or fluctuations in the incidence or prevalence of *Y. pestis* in rodent populations, we cannot predict when epizootics are likely to occur. Although the number of human plague cases in the prior year was not a significant predictor in our model, humans are only incidental hosts and the occurrence or absence of a major epizootic in the previous year could modify the expected relationship between rainfall and human cases. Because the amount of rainfall in June and July prior to the start of the plague season in August was a significant explanatory variable, the amount of lead time that the model provides is relatively limited. However, combined with improved rodent surveillance data, our model could be used to mobilize prevention and control efforts prior to the September to January peak in human plague cases. In addition, rainfall in East Africa is positively correlated with warm El Nino Southern Oscillation conditions and Pacific and Indian Ocean sea surface temperatures at a several month lag [5], [63], [64], [65], [66]. Further research is required to determine whether this association extends to the West Nile region of Uganda, where it could be used to predict elevated risk conditions with a longer lead time.

The spatial and temporal variation in vector-borne and zoonotic disease occurrence is often closely associated with climatic and environmental predictors, but the lack of reliable environmental and climatic data in many tropical and sub-tropical regions impedes our ability to model the association between these predictors and disease occurrence. More accurate models could improve our ability to target limited surveillance, prevention and control resources, because disease surveillance and reporting in these regions also tends to be sparse. In our study region, there was a wide range in accuracy among individual satellite, *in situ*, and simulated meteorological and climate datasets in representing regional rainfall variability. We have shown that ground-truthing these datasets (when possible) and the development of multi-dataset ensembles can be used to reduce the variability and uncertainty associated with individual meteorological datasets, and subsequently can generate more robust infectious disease model outcomes. Because the occurrence and risk of emergence of vector-borne and zoonotic infectious diseases is highest in tropical and sub-tropical regions where quality-controlled, ground-based meteorological data is often limited, an ensemble and model-averaging approach should often be an important component of disease modeling and forecasting efforts.

## Materials and Methods

### Study area

Previous studies have shown that the majority of the plague cases in the West Nile region of Uganda occur above 1300 m [35], [36]. Elevation in Vurra County ranges from 763–1573 m with a mean of 1140 m, and elevation in Okoro County ranges from 946–1873 m with a mean of 1458 m. The higher elevation areas are characterized by lush vegetation, fertile soils, numerous rivers and tributaries. The highlands in Okoro and Vurra also experience lower minimum and maximum temperatures and more annual rainfall than the Nile Valley region east of the escarpment (Figure 1b,c). Additional ecological characteristics of Okoro and Vurra counties, as well as the neighboring area outside of the plague focus, have previously been described in detail [36], [51], [56], [67], [68]. As of 2002, Okoro County had a population of 168,531 and Vurra County had a population of 98,412 (Uganda Bureau of Statistics, 2002).

### Epidemiological data

Epidemiological records of all suspected human plague cases within Arua and Zombo districts from 1999–2010 were compiled based on a review of health records from health clinics and hospitals [36]. Due to the scarcity of laboratory services in the study area, cases are generally identified as suspect based on clinical criteria. A suspect plague case is defined as rapid onset of fever, chills, headache, severe malaise, prostration with either (i) extremely painful swelling of lymph nodes in the arm-pits or inguinal area (bubonic plague), (ii) cough with blood-stained sputum, chest pain and difficult breathing in an area known to have plague or in a person who has in the recent past been to a plague-endemic area (pneumonic plague), or (iii) vomiting blood, bloody diarrhea, with or without history of contact with a known plague case, with or without history of a visit to a plague endemic area (septicemic plague). Case records included the place of residence, date of clinic visit, age, sex, and treatment. Because most plague cases occur in the home environment, we assume that exposure to *Y. pestis* occurred within a patient's county of residence. Of the 2490 suspected cases of plague identified from clinic records between 1 January 1999 and 31 July 2011, 995 (40.0%) occurred in Okoro County and 1414 (56.8%) in Vurra County. The remaining cases either originated in the DRC (42), Arua Municipality (1), or were of unknown origin (38), and were therefore excluded from analysis.

### Meteorological variables

Monthly mean temperatures and daily rainfall values for January 1999 to December 2010 were obtained for the Arua station from the handwritten weather logs archived at the airport, and subsequently digitized. A 15-month period of missing handwritten data from October 2000-December 2001 was in-filled using daily data for the Arua station that was archived by the National Oceanic and Atmospheric Administration's National Climate Data Center (NCDC). The NCDC record was not the first choice because the record, overall, is much less complete than the handwritten record provided by the airport and is subject to occasional transcription errors. The NCDC data was quality-controlled and outliers (>+/−4 standard deviations from the monthly means) were discarded. A small percentage of months still had some days with missing data. In these cases, monthly frequency data (i.e, days with rainfall >2 mm) was calculated by multiplying the ratio of the actual to measured days for the month. Because the Arua meteorological station is outside of our study area, has periods of questionable data due to collection techniques, and has several data gaps, we also selected several publicly available gridded gauge- and satellite-derived regional or global climate datasets from U.S. and European climate data archives (Table 1; Materials and Methods S1).

### Dataset ensembles

**Figure 6:**
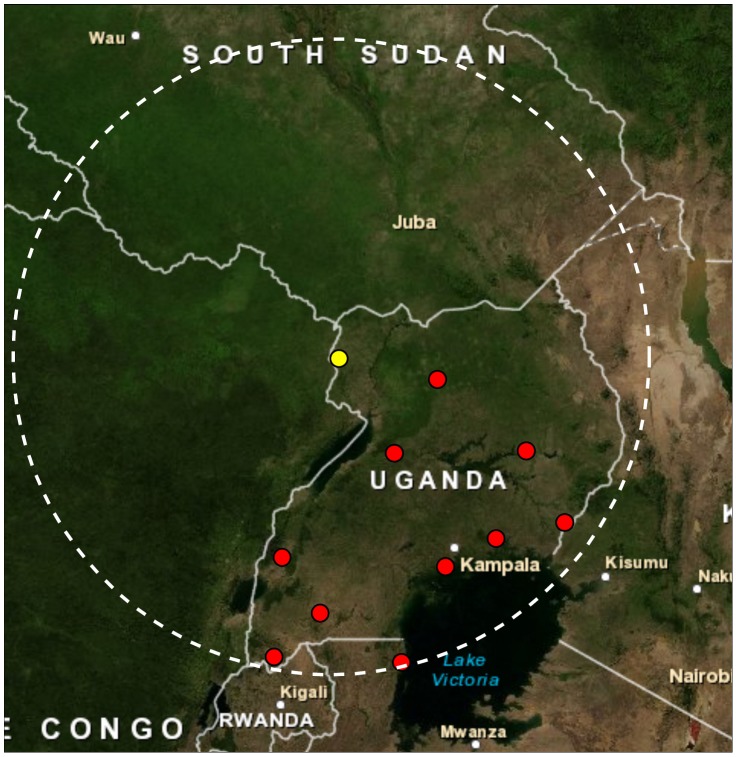
Map of meteorological stations within 500 km of Arua, Uganda. Meteorological stations containing rainfall data for at least 25% of the days between 1998 and 2010 (red circles) within 500 km of the Arua airport (yellow circle).

It is not possible to determine which temperature or rainfall datasets described above are most accurate for our study area due to the lack of local meteorological stations available for validation. To address the uncertainty in the accuracy of each dataset and the differences between the datasets (see Figure 4; Table S1), we considered individual datasets, as well as weighted dataset ensembles when constructing the statistical models of plague described below. For temperature we created an ensemble dataset for January, 1998 to December, 2010 by equally weighting the standardized monthly temperatures from the Arua and ERA-Interim datasets. For rainfall we considered five of the seven datasets individually (the ERA-Interim and NCEP datasets—which have the coarsest spatial resolution—had the lowest correlations with surrounding ground-based rainfall values and were therefore excluded; see Results) and an ensemble of all seven rainfall datasets. To create a rainfall dataset ensemble we first obtained daily rainfall totals for all meteorological stations within 500 km of Vurra and Okoro counties available from NCDC to ground-truth the spatial rainfall datasets. There were 11 stations within that radius that had rainfall totals for at least 25% of the days between 1999 and 2010 (Figure 6). For each station we calculated the number of days with greater than 0.2 mm of rainfall per season. When stations had missing data, seasonal frequency data was calculated by multiplying the ratio of the actual to measured days for the season. Seasonal rainfall frequencies for each station were standardized using the seasonal mean and variance from 2003–2010. We then calculated the correlation of the six rainfall datasets described in Table 1 with the standardized seasonal rainfall frequencies at each of the 11 meteorological stations. For each of the six datasets, this was done by extracting the rainfall frequencies from the grid point nearest each meteorological station, computing the correlation with the observed rainfall frequencies, and then computing an average correlation over the 11 stations. Finally, a weighted rainfall dataset ensemble was created by averaging all seven datasets each weighted by its average 11-station correlation coefficient. Because the Arua airport rainfall dataset is already a ground-based dataset and could not be independently validated, it was given an initial weight of 1/7 (0.14). To examine the sensitivity of our results to the weighting of this dataset we ranged its weight from 0.0 to 0.5 while holding proportional weighting of the other rainfall datasets constant (the results were found to be insensitive to the weighting of this dataset). For months where data was missing from a particular dataset (e.g. CMORPH prior to 2003), the ensemble average was re-weighted to exclude that dataset for the missing months only.

To reduce the number of potential explanatory variables, we grouped the rainfall data into four seasons and the temperature data into two seasons. The dry season runs from December to February, the secondary rainy season from March to May, the primary rainy season from August to November, with a rainy-season interval from June to July separating the two periods of heaviest rain (Figure 2b). The standardized numbers of days of rainfall per season in each category were considered as potential explanatory variables for the number of plague cases. Annual numbers of days of rainfall in each category were also included. Standardized mean monthly temperatures for August (coolest month) and February (warmest month) were included as potential explanatory variables, as was the standardized mean annual temperature. The standardized mean temperatures during the warm (January–April) and cool (June–October) seasons were also considered as potential explanatory variables (Figure 2c). The warm and cool seasons were defined as the months where the mean temperature was greater than 0.5 standard deviations above or below the annual mean temperature.

### Statistical analysis

Linear multiple regression models were constructed to identify meteorological predictors of annual plague case counts. The square root transformed number of suspect plague cases per year is normally distributed (Shapiro-Wilk test; W = 0.945, p = 0.58) and was used as the primary response variable. The monthly, seasonal, and annual rainfall and temperature variables described above were included as potential explanatory variables at both zero- and one-year lags. We defined the plague year as running from August to July of the following calendar year, but the annual rainfall and temperature variables were based on calendar year. The zero-year lags for the dry season (December–February), secondary rainy season (March–May), rainy-seasonal interval (June–July), and warm seasons (January–April) all occur prior to the start of the plague year in August. For example the March to May secondary rainy season in 1999 would be considered as a zero lag for the 1999–2000 plague year and as a one-year lag for the 2000–2001 plague year. Therefore the zero-year lags for monthly or seasonal variables prior to August could still be used to predict an upcoming plague year up to eight months in advance. Correspondingly, the primary rainy season from August to November does not occur prior to the start of the plague year.

The leaps package version 2.9 in R (Thomas Lumley 2009) was used to perform an exhaustive search of the best fit models with up to three explanatory variables with bias-adjusted Akaike's information criterion (AICc) as the model selection criterion (SI Materials and Methods). Model selection was performed using both the weighted meteorological ensemble dataset and each of the individual temperature and rainfall datasets. Once the most frequently included explanatory variables across these datasets were identified they were then used to determine (1) whether there was a consensus ‘best’ model across all meteorological datasets and (2) which variable coefficients remained statistically significant when averaged across all candidate models using model averaging techniques (SI Materials and Methods). Due to the limited size of the dataset, we were not able to withhold a portion of the data for model validation. Model sensitivity to the number of suspect plague cases in a given year was tested using the leave-one-out method where the analysis was rerun by sequentially dropping and then replacing each year from the model [69]. The leave-one-out method was used to create a mean and 95% confidence interval of the model r^2^ for comparison to the r^2^ from the best-fit model. All statistical analyses were performed in R 2.11 (R Development Core Team 2010).

## Supporting Information

Materials and Methods S1 Descriptions of temperature and precipitation datasets and model selection techniques.
(DOCX)

Table S1 Correlation matrix for the seven different rainfall datasets.
(DOCX)

Table S2 Correlation values for rainfall datasets at each of the 11 meteorological stations within a 500 km radius of our study region.
(DOCX)

Table S3 Mean model AICc, ΔAICc values and coefficient estimates and 95% confidence intervals for the consensus best two-variable models with square-root transformed number of suspect plague cases as the response variable.
(DOCX)
