# Supplementary material for: Improvement of Disease Prediction and Modeling through the Use of Meteorological Ensembles: Human Plague in Uganda
Source: PLoS One. 2012 Sep 14;7(9):e44431. doi: 10.1371/journal.pone.0044431 (PMC3443104; doi:10.1371/journal.pone.0044431)
Supplement: Materials and Methods S1 — Descriptions of temperature and precipitation datasets and model selection techniques. (DOCX) [file pone.0044431.s001.docx]

**Supporting Information**

**Additional Materials and Methods S1**

*Temperature datasets*

In addition to temperature data from the Arua airport, we also obtained monthly temperature data from the European Centre for Medium-Range Weather Forecasts Interim reanalysis project (ERA-Interim; Table 1), which assimilates multiple sources of surface, radar, and satellite data into a global operational forecast model to recreate past atmospheric and land-surface conditions from 1979-present [[1](#_ENREF_1)]. Mean monthly temperatures from both datasets were standardized using the monthly mean and variance from 2003–2010 (this time frame was selected based on the completeness of the temperature and rainfall datasets during this period). The standardized monthly mean temperatures from these two datasets were moderately correlated (r=0.59; Figure 4a), despite substantial differences in the areas that each dataset represents, which suggests both have reasonable representations of regional temperature variability.

*Rainfall datasets*

In addition to daily rainfall totals from Arua airport, six other gauge- and satellite-estimated or re-analysis rainfall datasets were selected based on their perceived accuracy in sub-Saharan Africa (Table 1). The Tropical Rainfall Measurement Mission (TRMM) Multisatellite Precipitation Analysis project [[2](#_ENREF_2)] combines microwave and infrared data from a variety of satellite instruments. The National Oceanic and Atmospheric Association Climate Prediction Center’s morphing technique (CMORPH) dataset combines precipitation estimates from low orbiting satellite microwave observations and transports the features using spatial propagation information from geostationary infrared satellite data [[3](#_ENREF_3)]. The ERA-Interim and the National Centers for Environmental Prediction-Department of Energy Reanalysis II (NCEP/DOE II; Kanamitsu et al. 2002) datasets assimilate multiple sources of surface, radar, and satellite data into a global operational forecast model to recreate past atmospheric and land-surface conditions from 1979-present. The Global Precipitation Climatology Project (GPCP) established by the World Climate Research Program also assimilates multiple data sources including gauge- and satellite-based instrumentation [[5](#_ENREF_5)]. The final rainfall dataset we included in our analyses was from the United States Agency for International Development’s (USAID) Famine Early Warning System Network (FEWS-NET), which uses satellite-based rainfall estimates to supplement data from the surface-based rain gauge network [[6](#_ENREF_6)]. The version employed here is a more temporally stable product, compiled by the NOAA Africa Rainfall Climatology project [[7](#_ENREF_7)]. Asadullah et al. [[8](#_ENREF_8)] evaluated five rainfall datasets including CMORPH, TRMM and FEWS-NET over Uganda and found that CMORPH and TRMM correlated most closely with station records of rainfall amount and occurrence. Consistent with our approach, Asadullah et al. [[8](#_ENREF_8)] suggested that more than one rainfall dataset should be used for any application due to the relative strengths and weaknesses of each.

Daily rainfall totals from each dataset were aggregated on a monthly basis by calculating the frequency of days per month where the amount of rainfall fell into the following overlapping categories: >0.2 mm, >2mm, between 0.2—10mm, between 2—10mm, >10mm, and >20mm. These frequency definitions allow us to examine how light, medium, heavy, extreme, and overall rainfall events influence the number of plague cases. Subsequently, the rainfall frequencies were standardized using the 3-month running mean and variance of the frequency data for 2003-2010 (the period for which we have data from all rainfall datasets but GPCP). The correlation between datasets in the standardized monthly days with rainfall >0.2mm varied widely (Table S1, Figure 4b). For example, the observational rainfall data from the Arua airport was moderately correlated with the TRMM and CMORPH data (r=0.57 for each), but only weakly correlated with the ERA-Interim and NCEP/DOE re-analysis data (r=0.22 and r=0.07). The strongest correlation was between the two satellite datasets, CMORPH and TRMM (r=0.71).

*Statistical analysis*

The leaps package version 2.9 in R (Thomas Lumley 2009) was used to perform an exhaustive search of the best fit models with up to three explanatory variables. However, none of the three-variable models had a statistically significant model-averaged third variable, so we limited our analysis to models with two or fewer explanatory variables. Bias-adjusted Akaike’s information criterion (AICc) was used as the model selection criterion to correct for the small sample size [[9](#_ENREF_9)].

The best model is the one with the smallest AICc (AICc*_min_*), and all other models are then compared to the best model by calculating their AICc difference ∆*_i_ =* AICc*_i_* - AICc*_min_*, where *i* represents a particular model. Models with ∆*_i_* values of less than 2 are considered to have substantial support, while models with ∆*_i_ >* 10 are considered to have almost no support [[9](#_ENREF_9)]. Several of the meteorological variables were highly correlated, so the predictors in the model were tested for multicollinearity by comparing variance inflation factors (VIF). All models with a VIF > 5 were excluded from analysis.

Model selection was performed using all 18 possible combinations of the 3 individual and ensemble temperature datasets and 6 individual and ensemble rainfall datasets. All models with a ΔAICc < 2 for a particular rainfall and temperature dataset combination were considered as potential candidate models. The frequency with which each temperature or rainfall variable was included in these candidate models was calculated to identify the most likely explanatory variables. Once the most frequently included explanatory variables were identified they were then used to determine (1) whether there was a consensus ‘best’ model across all meteorological datasets and (2) which variable coefficients remained statistically significant when averaged across all candidate models.

First, the most frequently included explanatory variables were used to select several leading two-variable models from among the list of potential candidate models. These leading models were then run with each of the 18 rainfall and temperature dataset combinations. The mean and variance of each model’s adjusted r^2^ across these datasets was used to select a consensus ‘best’ model or models. Coefficient estimates for each variable were calculated using a Bayesian model averaging approach that incorporates both within-dataset variance and between-dataset variance [[10](#_ENREF_10)]. The coefficient estimates for each leading model ($\hat{\theta}_{i})$were averaged across the estimates from each of the (*R*=18) dataset combinations with each given equal weight (*w_i_*=1/18) as a conservative estimate. The model-averaged coefficients estimates have a mean of $\hat{\overline{\theta}}=\sum_{i=1}^{R} (w_{i}\hat{\theta}_{i})$ with a variance of $var\left( \hat{\overline{\theta}} \right)=\sum_{i=1}^{R} w_{i}\cdot\left[ var\left( \hat{\theta} | g_{i} \right)+\left( \hat{\theta}_{i}-\hat{\overline{\theta}} \right)^{2} \right]$, where $var\left( \hat{\theta} | g_{i} \right)$ represents the variance for a particular dataset *g_i_*, and $\left( \hat{\theta}_{i}-\hat{\overline{\theta}} \right)^{2}$ incorporates the between-dataset variance.

**References**

1. Dee DP, Uppala SM, Simmons AJ, Berrisford P, Poli P, et al. (2011) The ERA-Interim reanalysis: configuration and performance of the data assimilation system. Quarterly Journal of the Royal Meteorological Society 137: 553-597.

2. Huffman GJ, Adler RF, Bolvin DT, Gu GJ, Nelkin EJ, et al. (2007) The TRMM multisatellite precipitation analysis (TMPA): Quasi-global, multiyear, combined-sensor precipitation estimates at fine scales. Journal of Hydrometeorology 8: 38-55.

3. Joyce RJ, Janowiak JE, Arkin PA, Xie PP (2004) CMORPH: A method that produces global precipitation estimates from passive microwave and infrared data at high spatial and temporal resolution. Journal of Hydrometeorology 5: 487-503.

4. Kanamitsu M, Ebisuzaki W, Woollen J, Yang SK, Hnilo JJ, et al. (2002) Ncep-Doe Amip-Ii Reanalysis (R-2). Bulletin of the American Meteorological Society 83: 1631-1643.

5. Huffman GJ, Adler RF, Morrissey MM, Bolvin DT, Curtis S, et al. (2001) Global precipitation at one-degree daily resolution from multisatellite observations. Journal of Hydrometeorology 2: 36-50.

6. Xie PP, Arkin PA (1996) Analyses of global monthly precipitation using gauge observations, satellite estimates, and numerical model predictions. Journal of Climate 9: 840-858.

7. Love TB, Kumar V, Xie PP, Thiaw W. A 20-year daily Africa precipitation climatology using satellite and gauge data; 2004; Seattle, WA. Amer. Meteor. Soc.

8. Asadullah A, McIntyre N, Kigobe M (2008) Evaluation of five satellite products for estimation of rainfall over Uganda. Hydrological Sciences Journal-Journal Des Sciences Hydrologiques 53: 1137-1150.

9. Burnham KP, Anderson DR (2002) Model selection and multimodel inference : a practical information-theoretic approach. New York: Springer. xxvi, 488 p. p.

10. Johnson JB, Omland KS (2004) Model selection in ecology and evolution. Trends in Ecology & Evolution 19: 101-108.
