# Supplementary material for: Improvement of Disease Prediction and Modeling through the Use of Meteorological Ensembles: Human Plague in Uganda
Source: PLoS One. 2012 Sep 14;7(9):e44431. doi: 10.1371/journal.pone.0044431 (PMC3443104; doi:10.1371/journal.pone.0044431)
Supplement: Table S1 — Correlation matrix for the seven different rainfall datasets. (DOCX) [file pone.0044431.s002.docx]

Table S1. Correlation matrix for the seven different rainfall datasets.

|  | Arua Obs. | CMORPH | TRMM | FEWS-Net | GPCP | ERA-Interim | NCEP/DOE |
| --- | --- | --- | --- | --- | --- | --- | --- |
| Arua Obs | - | 0.569 | 0.574 | 0.432 | 0.382 | 0.216 | 0.072 |
| CMORPH |  | - | 0.717 | 0.593 | 0.572 | 0.488 | 0.042 |
| TRMM |  |  | - | 0.509 | 0.494 | 0.246 | 0.270 |
| FEWS-Net |  |  |  | - | 0.297 | 0.305 | 0.049 |
| GPCP |  |  |  |  | - | 0.422 | 0.150 |
| ERA-Interim |  |  |  |  |  | - | -0.053 |
| NCEP/DOE |  |  |  |  |  |  | - |

Correlation values are based on the standardized monthly frequencies of >0.2mm of rainfall (see text for details).
