# Supplementary material for: Improvement of Disease Prediction and Modeling through the Use of Meteorological Ensembles: Human Plague in Uganda
Source: PLoS One. 2012 Sep 14;7(9):e44431. doi: 10.1371/journal.pone.0044431 (PMC3443104; doi:10.1371/journal.pone.0044431)
Supplement: Table S2 — Correlation values for rainfall datasets at each of the 11 meteorological stations within a 500 km radius of our study region. (DOCX) [file pone.0044431.s003.docx]

Table S2. Correlation values for rainfall datasets at each of the 11 meteorological stations within a 500 km radius of our study region.

| Dataset | Arua | Gulu | Masindi | Entebbe | Soroti | Tororo | Jinja | Mbarara | Kasese | Kabale | Bukoba | Mean |
| --- | --- | --- | --- | --- | --- | --- | --- | --- | --- | --- | --- | --- |
| CMORPH | 0.18 | 0.74 | -0.27 | -0.17 | 0.33 | 0.13 | 0.30 | 0.16 | 0.27 | -0.25 | 0.34 | 0.16 |
| TRMM | 0.40 | 0.34 | 0.04 | 0.31 | 0.26 | -0.45 | 0.59 | 0.24 | 0.21 | 0.12 | 0.49 | 0.23 |
| FEWS-Net | 0.40 | 0.34 | 0.02 | 0.24 | 0.57 | 0.25 | 0.30 | 0.41 | 0.28 | 0.15 | 0.80 | 0.34 |
| GPCP | 0.36 | -0.38 | -0.15 | 0.13 | 0.36 | 0.11 | 0.46 | 0.19 | 0.29 | 0.05 | 0.47 | 0.17 |
| ERA-Interim | 0.00 | 0.33 | -0.20 | 0.03 | 0.21 | 0.12 | 0.11 | 0.10 | 0.14 | -0.13 | 0.21 | 0.08 |
| NCEP/DOE | 0.15 | -0.29 | 0.07 | 0.06 | 0.08 | -0.03 | 0.11 | 0.16 | 0.00 | 0.06 | 0.56 | 0.08 |
| **Mean** | 0.25 | 0.18 | -0.08 | 0.10 | 0.30 | 0.02 | 0.31 | 0.21 | 0.20 | 0.00 | 0.48 | 0.18 |

Correlation values are based on standardized seasonal frequencies of >0.2mm rainfall (see text for details).
