# Supplementary material for: Improvement of Disease Prediction and Modeling through the Use of Meteorological Ensembles: Human Plague in Uganda
Source: PLoS One. 2012 Sep 14;7(9):e44431. doi: 10.1371/journal.pone.0044431 (PMC3443104; doi:10.1371/journal.pone.0044431)
Supplement: Table S3 — Mean model AICc, ΔAICc values and coefficient estimates and 95% confidence intervals for the consensus best two-variable models with square-root transformed number of suspect plague cases as the response variable. (DOCX) [file pone.0044431.s004.docx]

Table S3. Mean model AICc, ∆AICc values and coefficient estimates and 95% confidence intervals for the consensus best two-variable models with square-root transformed number of suspect plague cases as the response variable.

| ***Model*** | **Coefficient** | **AICc** | **∆AICc** | **Estimate** | **95% CI** |
| --- | --- | --- | --- | --- | --- |
| *Model #1* | | 26.6 | 0 |  |  |
|  | **Intercept** |  |  | **12.01** | **9.56 — 14.46** |
|  | **>2mm Dry season rainfall** |  |  | **-1.95** | **-3.41 — -0.49** |
|  | **0.2-10mm June/July rainfall** |  |  | **1.76** | **0.06 — 3.46** |
| *Model #2* | | 28.4 | 1.8 |  |  |
|  | **Intercept** |  |  | **12.41** | **9.88 — 14.94** |
|  | **>10mm Dry season rainfall** |  |  | **-2.05** | **-3.87 — -0.23** |
|  | **0.2-10mm June/July rainfall** |  |  | **2.19** | **0.17 — 4.21** |
| *Model #3* | | 28.7 | 2.1 |  |  |
|  | **Intercept** |  |  | **11.84** | **9.32 — 14.36** |
|  | **>2mm Dry season rainfall** |  |  | **-2.04** | **-3.49 — -0.59** |
|  | >2mm June/July rainfall |  |  | 1.50 | -0.16 — 3.17 |
| *Model #4* | | 31.1 | 4.5 |  |  |
|  | **Intercept** |  |  | **12.32** | **9.60 — 15.05** |
|  | **>10mm Dry season rainfall** |  |  | **-2.00** | **-3.87 — -0.13** |
|  | **>2mm June/July rainfall** |  |  | **1.95** | **0.06 — 3.85** |
| *Model #5* | | 32.1 | 5.5 |  |  |
|  | **Intercept** |  |  | **11.52** | **8.02 — 15.02** |
|  | **>2mm Dry season rainfall** |  |  | **-1.99** | **-3.79 — -0.19** |
|  | >20mm Dry season rainfall – one year lag |  |  | 0.02 | -3.63 — 3.67 |

∆AICc represents the difference between a model’s mean AICc value and the mean AICc value of the best fit overall model. Bold text indicates coefficients significant at the α=0.05 level.
